# Sex-Specific Age-Related Changes in Excitatory and Inhibitory Intracortical Circuits in Mouse Primary Auditory Cortex

**DOI:** 10.1523/ENEURO.0378-24.2024

**Published:** 2025-02-03

**Authors:** Zheng Xu, Binghan Xue, Joseph P. Y. Kao, Patrick O. Kanold

**Affiliations:** ^1^Department of Biomedical Engineering, Johns Hopkins University, Baltimore, Maryland 20215; ^2^Department of Biology, University of Maryland, College Park, Maryland 20742; ^3^Center for Biomedical Engineering and Technology, and Department of Physiology, University of Maryland School of Medicine, Baltimore, Maryland 21201

**Keywords:** aging, excitation, GABA, inhibition, primary auditory cortex, translaminar circuits

## Abstract

A common impairment in aging is age-related hearing loss (presbycusis), which manifests as impaired spectrotemporal processing. Presbycusis can be caused by a dysfunction of the peripheral and central auditory system, and these dysfunctions might differ between the sexes. To date, the circuit mechanisms in the central nervous system responsible for age-related auditory dysfunction remain mostly unknown. In the auditory cortex (ACtx), aging is accompanied by alteration in normal inhibitory (GABA) neurotransmission and changes in excitatory (NMDA and AMPA) synapses, but which circuits are affected has been unclear. Here we investigated how auditory cortical microcircuits change with age and if sex-dependent differences existed. We performed laser-scanning photostimulation (LSPS) combined with whole-cell patch-clamp recordings from layer (L) 2/3 cells in the primary auditory cortex (A1) in young adult (2–3 months) and aged (older than 18 months) male and female CBA/CaJ mice which have normal peripheral hearing. We found that L2/3 cells in aged male animals display functional hypoconnectivity of inhibitory circuits originating from L2/3 and L4. Compared with cells from young adult mice, cells from aged male mice have weaker excitatory connections from L2/3. We also observed an increased diversity of excitatory and inhibitory inputs. These results suggest a sex-specific reduction and diversification in excitatory and inhibitory intralaminar cortical circuits in aged mice compared with young adult animals. We speculate that these unbalanced changes in cortical circuits contribute to the functional manifestations of age-related hearing loss in both males and females.

## Significance Statement

A common impairment in aging is age-related hearing loss (presbycusis) which can be caused by a dysfunction of the peripheral and central auditory system. Impaired spectrotemporal processing in the auditory cortex is thought to underlie some of these impairments. We compare auditory cortex circuits in vitro in young adult (∼P60) and aged (∼P585) CBA mice. We find a sex-specific reduction in excitatory and inhibitory intralaminar cortical circuits in aged mice compared with young adult animals. We speculate that these unbalanced changes in cortical circuits underlie the differential functional manifestations of age-related hearing loss in both males and females.

## Introduction

Sensory and cognitive declines with aging are profound and likely involve changes in cortical processing (Nusbaum, 1999; Scialfa, 2002; Jayakody et al., 2018). One common impairment is age-related hearing loss, also known as presbycusis (Brant and Fozard, 1990; Pearson et al., 1995; Matthews et al., 1997; Cruickshanks et al., 1998). Both peripheral degeneration of the auditory transduction mechanisms in the ear (Gopinath et al., 2009; Lin et al., 2011) and changes in the central auditory areas that further process those peripheral signals (Caspary et al., 2008; Cisneros-Franco et al., 2018) contribute to hearing difficulty with aging. Peripheral changes leading to hearing loss have been well studied, but less is known about the central auditory changes with aging. These central changes might contribute to a large proportion of hearing difficulties, as aging humans with intact auditory periphery still perform worse on auditory tasks (Fitzgibbons and Gordon-Salant, 1998; Gordon-Salant and Fitzgibbons, 1999; Lister et al., 2002; Wambacq et al., 2009). Moreover, age-dependent decreases are sex-dependent, with larger decreases in hearing present in males than females (Pearson et al., 1995; Cruickshanks et al., 1998; Helzner et al., 2005; Nolan, 2020; Hansen et al., 2024). In animal models, age-related loss of high-frequency hearing has been associated with changes in tonotopic organization and deterioration of temporal processing in the inferior colliculus (IC) and auditory cortex (ACtx; Willott et al., 1993; Mendelson and Ricketts, 2001; Lee et al., 2002; Mendelson and Lui, 2004; Gourevitch and Edeline, 2011; Engle and Recanzone, 2013; Trujillo et al., 2013; Trujillo and Razak, 2013; Brewton et al., 2016; Recanzone, 2018; Shilling-Scrivo et al., 2021). Moreover, an abnormal tuning bandwidth has been observed in aging rats (Turner et al., 2005). In vivo imaging of the primary auditory cortex (A1) in mice of the CBA strain, which retains good peripheral hearing into adulthood (Willott et al., 1988, 1991; Bowen et al., 2020), has shown that aging leads to a reduction in the diversity of tuning curves, a lack of suppressive responses, increased correlated activity (Shilling-Scrivo et al., 2021), and altered behavioral modulation of sound-evoked activity (Shilling-Scrivo et al., 2022). These functional changes are likely due to altered functional connections between A1 neurons. In these animal studies, the age-dependent changes were more pronounced in males than females, suggesting that central age-dependent changes might be more prominent in males than those in females (Shilling-Scrivo et al., 2021, 2022; Mittelstadt et al., 2024).

The interplay of glutamatergic excitation and GABAergic inhibition contributes to typical brain function. Disruption of inhibition is thought to be responsible for age-related impairment of suprathreshold signals including auditory signal in noise (Chao and Knight, 1997; Kok, 1999; Caspary et al., 2008; Stanley et al., 2012; Rozycka and Liguz-Lecznar, 2017; Recanzone, 2018; Shilling-Scrivo et al., 2021). Indeed, aging decreases the density of inhibitory synapses in the prefrontal cortex (Peters et al., 2008). It is also associated with decreased levels of glutamate decarboxylase (GAD) and vesicular GABA transporter (VGAT), hypofunction of NMDA receptors, and changes in ionic conductances in different sensory cortices (De Luca et al., 1990; Chaudhry et al., 1998; Milbrandt et al., 2000; Shi et al., 2004; Stanley and Shetty, 2004; Ling et al., 2005; Burianova et al., 2009; Stanley et al., 2012; Gold and Bajo, 2014; Liguz-Lecznar et al., 2015; Liao et al., 2016; Kumar et al., 2019). Interestingly, administering GABA or GABA agonists facilitates visual function in vision-impaired aged animals (Leventhal et al., 2003). Similarly, GABA-associated changes are observed in the auditory pathway in aged animals. Aging alters GABA receptor composition, the levels of GAD, and calcium-binding proteins in the IC, thalamus, cochlear nucleus, and ACtx (Milbrandt et al., 1996; Caspary et al., 1999; Ling et al., 2005; Ouda and Syka, 2012; Caspary et al., 2013; Richardson et al., 2013; Brewton et al., 2016; Recanzone, 2018; Richardson et al., 2020; Rogalla and Hildebrandt, 2020).

These results suggest that the primary functional deficit in cortical circuits in aging may lie in a hypofunction of inhibitory circuits. At the same time, however, synaptic excitation also changes with aging, since the density of dendritic spines is decreased in aged brains and the structure of the NMDA receptor complex changes with aging (Magnusson et al., 2010; Dickstein et al., 2013; Hickmott and Dinse, 2013; Fetoni et al., 2015). These results suggest that both excitatory and inhibitory circuits may be altered in aged brains. Changes in functional microcircuits could contribute to the observed changes in sound processing; e.g., increased convergence of connections across the tonotopic axis could contribute to changes in bandwidth (Willott et al., 1993; Mendelson and Ricketts, 2001; Lee et al., 2002; Mendelson and Lui, 2004; Engle and Recanzone, 2013; Brewton et al., 2016; Recanzone, 2018; Richardson et al., 2020; Shilling-Scrivo et al., 2021) and an increase in population correlations of cortical neurons (Shilling-Scrivo et al., 2021). However, it is unknown if the functional connectivity of microcircuits in A1 changes in aging and if there are sex-dependent differences in how microcircuits change with age.

We thus investigated the changes in functional microcircuits of A1 with aging. Mice of the C57BL/6J strains have peripheral hearing loss in young adulthood (Henry and Chole, 1980; Willott, 1986; Spongr et al., 1997) and show a remapping of ACtx in response to peripheral hearing loss (Willott et al., 1993). In these mice, both intracortical excitatory and inhibitory circuits in ACtx are altered (Xue et al., 2023). Here, we were interested in identifying the effects of aging on A1. We, therefore, used a model that has an intact peripheral auditory system. CBA mice are one such model, exhibiting good peripheral hearing into old age (Willott et al., 1988, 1991; Frisina et al., 2011; Bowen et al., 2020; Shilling-Scrivo et al., 2021) and thus allow us to investigate the circuit effects of aging on A1 neurons. We used laser-scanning photostimulation (LSPS) in combination with whole-cell patch-clamp recordings of A1 L2/3 cells in young and old CBA mice. Overall, our findings reveal a specific age-related hypoconnectivity of intracortical excitatory and inhibitory circuits from L4 in A1. The nature of the hypoconnectivity differed between males and females. Our results thus suggest that not all circuits are changing equally and that there are sex differences. Therefore, therapeutic interventions, e.g., training paradigms, must take these laminar and sex-specific changes into account.

## Materials and Methods

All procedures were approved by Johns Hopkins University Institutional Animal Care and Use Committee.

### Animals

Male and female CBA/CaJ mice (Jackson Laboratory #000654) were raised in 12 h light/dark light cycle (*N* = 17 adult group, 2–3 months old; *N* = 21 aging group, 18–24 months old).

### Slice preparation

**Figure 1.**
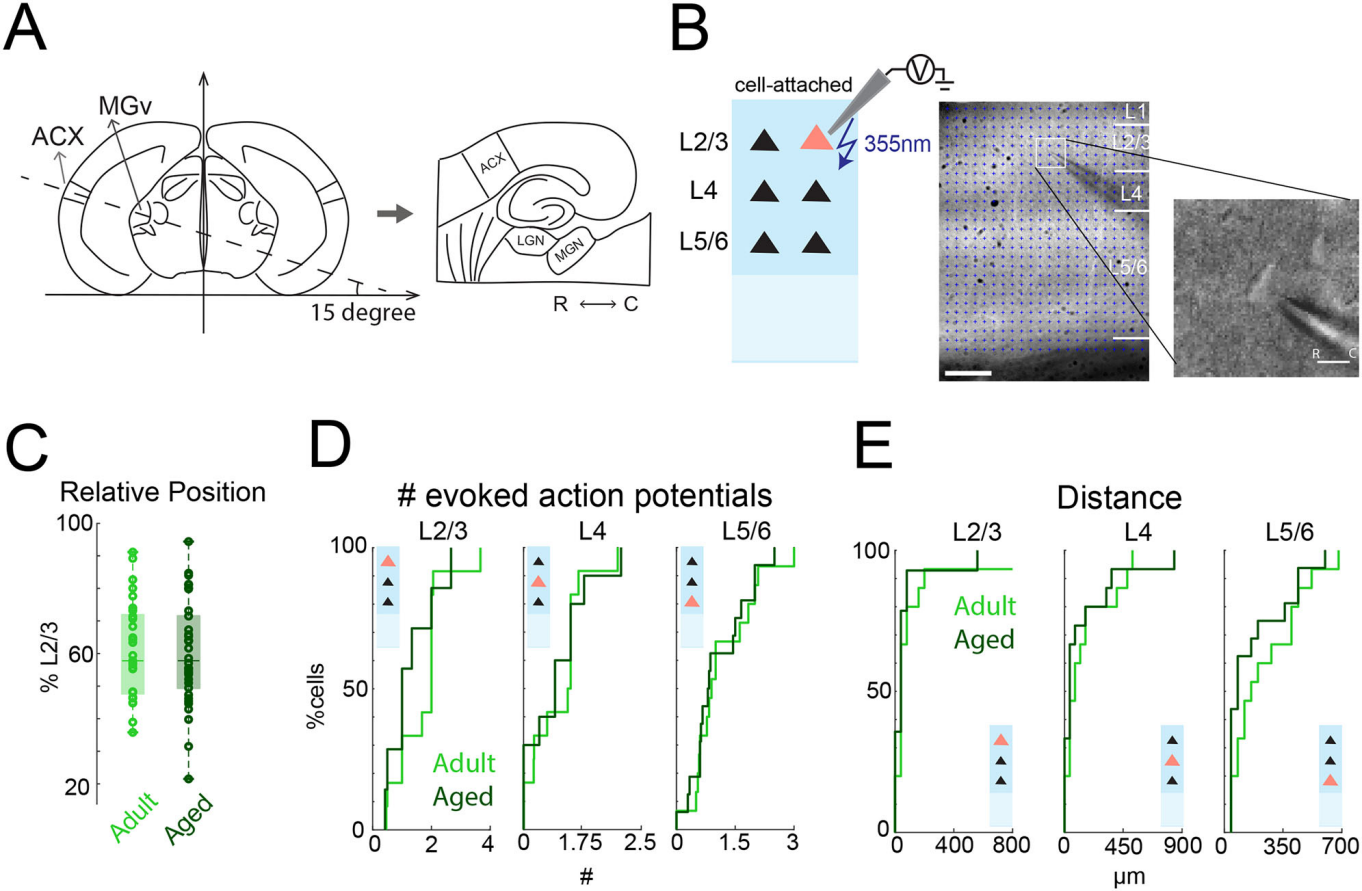
Photoexcitability of A1 neurons remains unchanged during aging. ***A***, Schematic of thalamocortical slicing. Slices were cut ∼15° from the horizontal plane to maintain the tonotopic organization in the slice. ***B***, Left, Schematic of LSPS for cell-attached recording. Solid pink triangle represents recorded neuron. Middle, Infrared image of brain slice with patch pipette on L2/3 neuron. Stimulation grid is indicated by blue dots. Layer boundaries are indicated by white bars on the right. The scale bar at the bottom left is 200 μm. Right, Magnified infrared image with pipette patching a L2/3 pyramidal neuron. The scale bar at the bottom right is 15 μm. ***C***, Relative position of recorded neurons within L2/3 relative to the borders of L4 and L1 (100%). Cells were sampled from a similar area in the middle of L2/3 in young adult and aged animals. ***D***, ***E***, Number of evoked action potentials (***C***) and effective stimulation distance (***D***) from cell-attached recordings of L2/3, L4 and L5/6 neurons. The numbers of evoked action potentials of neurons in all layers were similar and most spikes were evoked within 150 μm.

Mice were deeply anesthetized with isoflurane (Halocarbon). A block of brain containing A1 and the medial geniculate nucleus (MGN) was removed, and slices (400 μm thick) were cut on a vibrating microtome (Leica) in ice-cold artificial cerebrospinal fluid (ACSF) containing the following (in mM): 130 NaCl, 3 KCl, 1.25 KH_2_PO_4_, 20 NaHCO_3_, 10 glucose, 1.3 MgSO_4_, and 2.5 CaCl_2_, pH 7.35–7.4 (in 95% O_2_/5% CO_2_). Slices were cut ∼15° from the horizontal plane to maintain the tonotopic organization in the slice (Fig. 1*A*; Cruikshank et al., 2002; Zhao et al., 2009; Meng et al., 2015, 2017a,b). Left hemisphere slices were incubated for 1 h in ACSF at 30°C and then kept at room temperature. For recording, slices were held in a chamber on a fixed-stage microscope (Olympus BX51) and superfused (2–4 ml/min) with high-Mg recording solution at room temperature to reduce spontaneous activity in the slice. The high-Mg recording solution contained the following (in mM): 124 NaCl, 5 KCl, 1.23 NaH_2_PO_4_, 26 NaHCO_3_, 10 glucose, 4 MgCl_2_, and 4 CaCl_2_. The location of the recording site in A1 was identified by landmarks (Cruikshank et al., 2002; Zhao et al., 2009; Meng et al., 2015).

### Electrophysiology

Whole-cell recordings were performed with a patch-clamp amplifier (MultiClamp 700B, Molecular Devices) using pipettes with input resistance of 4–9 MΩ. Pyramidal cells were targeted based on their morphology (Fig. 1*B*). Pyramidal cells targeted for recording were in an area of A1 overlying the rostral flexure of the hippocampus. Data acquisition was performed by National Instruments AD boards and custom software (Ephus; Suter et al., 2010), written in MATLAB (MathWorks) and adapted to our setup. Voltages were corrected for an estimated junction potential of 10 mV. Electrodes were filled with an internal solution containing the following (in mM): 115 cesium methanesulfonate (CsCH_3_SO_3_), 5 NaF, 10 EGTA, 10 HEPES, 15 CsCl, 3.5 MgATP, and 3 QX-314, pH 7.25 (300 mOsm). Biocytin or neurobiotin (0.5%) was added to the electrode solution as needed. Series resistances were typically 20–25 MΩ.

### Laser-scanning photostimulation

LSPS was performed as described previously (Meng et al., 2015, 2017a,b, 2020). Caged glutamate [0.8 mM *N*-(6-nitro-7-coumarylmethyl)-ʟ-glutamate; Muralidharan et al., 2016] was added to the high-divalent ACSF during recording. Laser stimulation (1 ms) was delivered through a 10× water immersion objective (Olympus). Laser power on the specimen was <25 mW and was held constant between recordings. The power was chosen based on prior experiments resulting in reliable activation previously (Meng et al., 2015, 2017a,b, 2020). We performed cell-attached recordings to confirm this in aged mice (Fig. 1). For each map, an array of up to 30 × 30 sites with 40 μm spacing was stimulated once at 1 Hz in a pseudorandom order. This stimulation paradigm evokes an action potential at the stimulation sites with similar spatial resolution (∼100 μm) over cells in all cortical layers (Meng et al., 2015, 2017a,b). Thus, we spatially oversample at ∼3× in each dimension. Putative monosynaptic excitatory postsynaptic currents (EPSCs) were classified by the poststimulation latency of the evoked current. Evoked currents with latencies of <8 ms are likely to be the result of direct activation of glutamate receptors on the patched cell. Evoked currents with latencies between 8 and 50 ms were classified as monosynaptic evoked EPSCs. The first peak amplitude and the charge (the area of EPSC in the counting window) were quantified for each synaptic response. Recordings were performed at room temperature and in high-Mg^2+^ solution to reduce the probability of polysynaptic inputs. Cells that did not show any large (>100 pA) direct responses were excluded from the analysis, as these could be astrocytes. Excitatory and inhibitory inputs were recorded at −70 and 0 mV, respectively.

### Data analysis

Data were analyzed blind and semiautomated by custom software written in MATLAB. Cortical layer boundaries were identified by features in the bright-field image, e.g., cell density, cell body shape, and opacity, as described previously (Meng et al., 2015, 2017a,b, 2020). For each cell, we identified stimulus locations that gave rise to an evoked PSC and generated a binary input map for EPSCs and IPSCs. For each group, we aligned all individual maps to the soma position of the individual cells and averaged them. The resulting maps represent the spatial connection probability maps. For each cell we measured parameters of the laminar inputs from the individual connection maps. The input area was calculated from the binary input map as the area (numbers of pixels multiplied by pixel size) within each layer that gave rise to PSCs. The integration distance was calculated by first summing the amplitude of all inputs within a layer for each location across the rostrocaudal direction. We then measured the distance that covered 80% of evoked PSCs along the rostrocaudal direction. The mean charge was calculated as the average charge of the PSCs from each stimulus location. The mean peak amplitude was calculated as the average amplitude of PSCs at each stimulus location. For each cell, the balance of excitation and inhibition was calculated as the *E*/*I* ratio, which is based on the number of inputs and the average input strength (*E*_density_/*I*_density_ and *E*_charge_/*I*_charge_) in each layer.

To determine the diversity of connection patterns in each group, we calculated the spatial correlation of the binary connection maps of cells in each group by calculating the pairwise cross-correlations (Meng et al., 2020). For pairwise correlation calculation, we set the area that has monosynaptic connections to 1 and the area without monosynaptic connections to 0. For each cell, we derive a 2D matrix containing 0 and 1, which represent no connection spots and connection spots, respectively. We then apply corrcoef function in MATLAB to obtain the correlation value between cells. Higher correlation suggests more similar connection patterns and lower correlation indicates greater heterogeneity of cortical circuits. The correlation coefficient is calculated based on the following formula:

ρ(A,B)=cov(A,B)/σAσB.

Statistics results are plotted as means ± SD unless otherwise indicated. Populations combining both males and females, denoted by adult all and aged all, are tested for normality first using the Shapiro–Wilk (SW) test and compared with Student’s *t* test or rank-sum test accordingly. Populations that are sex specified, denoted by adult females, adult males, aged females, and aged males, are tested for normality first using the Shapiro–Wilk (SW) test and compared with a one-way ANOVA test and Kruskal–Wallis test accordingly. If at least one layer of the same measuring variables did not pass the normality test, rank-sum test was used for the comparison between adult all and aged all, and the Kruskal–Wallis test was used for the comparison among adult females, adult males, aged females, and aged males. In addition, the Kolmogorov–Smirnov test is used for comparison in cumulative distribution function(cdf) plots.

## Results

To investigate the changes of intracortical circuits in A1 with aging, we performed LSPS, as described in our prior studies (Meng et al., 2015, 2017a,b, 2020), in aged (older than 18 months) and young adult (2–3 months) CBA mice. Thalamocortical slices of A1 (Fig. 1*A*) were cut, and LSPS with caged glutamate was used to focally activate cortical neurons. Cell-attached patch recordings were performed to test the photoexcitability of A1 neurons, and whole-cell recordings were performed to test the spatial connectivity of excitatory and inhibitory inputs to L2/3 pyramidal neurons in A1.

### Aging does not alter photoexcitability of A1 neurons

To reliably compare the spatial connection pattern of cells in A1 using LSPS, we needed to confirm that the spatial resolution of LSPS was similar across ages. We performed cell-attached patch recordings with LSPS in cells (91 cells) from L2/3 (*n* = 15/15 cells in adult/aged), L4 (*n* = 15/15 cells in adult/aged), and L5/6 (*n* = 15/16 cells in adult/aged) to test the ability of A1 neurons to fire action potentials in response to photoreleased glutamate (*N* = 4, adult; *N* = 3 aged mice). Recorded cells were identified as pyramidal based on their appearance under illumination and were located at similar laminar positions across groups (Fig. 1*B*; *p* > 0.05). Short UV laser pulses (1 ms) were targeted to multiple stimulus locations to focally release glutamate and cause firing of action potentials. The grid of stimulation spots covered all layers within the slice, resulting in a high-resolution 2D photoactivation pattern for a given cell. We then counted numbers of evoked action potentials and measured the distance of effective stimulation sites. The numbers of evoked spikes were similar across ages (Fig. 1*C*; *p* > 0.05). Furthermore, the majority of action potentials were generated within 150 μm of the soma in both control and aged animals (Fig. 1*D*; *p* > 0.05). These findings suggest that the sensitivity of cells in all layers to photoreleased glutamate and thus the spatial resolution of LSPS remains unchanged with age.

### L2/3 neurons in aged A1 show sex-specific reduction in intralaminar excitatory connections

**Figure 2.**
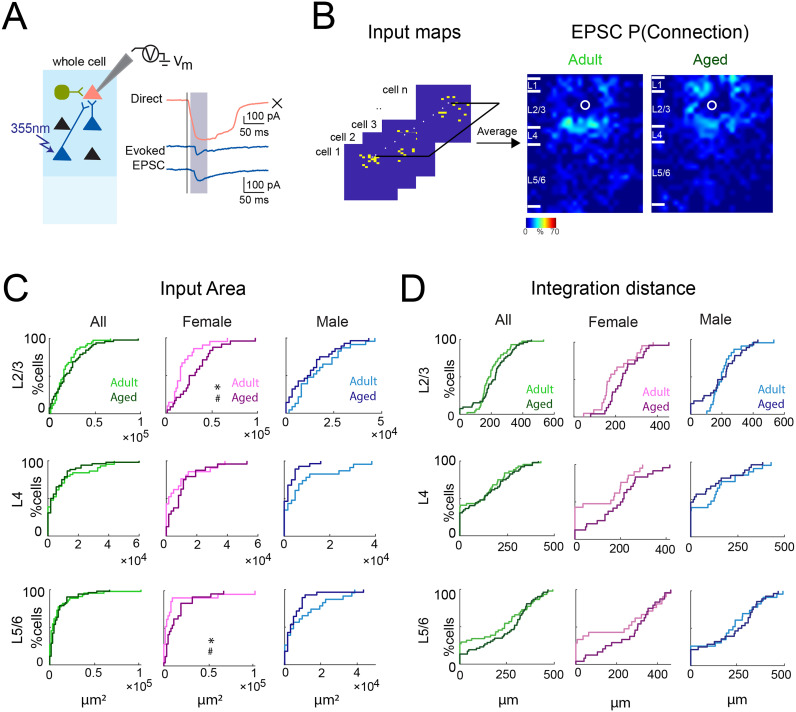
Sex-specific changes in excitatory connections to A1 L2/3 neurons in aged male mice. ***A***, Schematic of LSPS when recording evoked EPSCs. Whole-cell voltage-clamp recordings were obtained from pink cell at a holding potential of −70 mV (EPSCs). If the presynaptic excitatory neuron (e.g., yellow cells) synapses on the recorded neuron, an EPSC will be observed. Shown on the right are examplar patch-clamp recordings of direct response (pink) and EPSCs (yellow), acquired at the holding potential of −70 mV. Black vertical line indicates time of photostimulation; shaded gray box labels the time window of evoked responses. ***B***, Left, Schematic illustration of how the connection probability map is calculated. Connection maps from recorded neurons are aligned to the soma location and averaged to create the connection probability map. Right, Maps of connection probability for excitatory connections in young adult (left) and aged (right) animals. Soma location is indicated by the white circle. Connection probability is encoded according to the pseudocolor scale. White horizontal lines indicate averaged laminar borders and are 100 μm long. ***C***, Left, Distributions of the area of excitatory input originating from L2/3 (top), L4 (middle), and L5/6 (bottom) of young adult (light green) or aged (dark green) animals. Middle, Distributions of the area of excitatory input originating from L2/3 (top), L4 (middle), and L5/6 (bottom) of adult female (light pink) or aged female (dark pink) animals. Right, Distributions of the area of excitatory input originating from L2/3 (top), L4 (middle), and L5/6 (bottom) of young adult male (light blue) or aged male (dark blue) animals. *p_KS_ < 0.05; ^#^*p*_Kruskal–Wallis_ < 0.05. ***D***, Left, Integration distance to each L2/3 cell originating from L2/3 (top), L4 (middle), and L5/6 (bottom) of young adult or aged animals. Middle, Integration distance to each L2/3 cell originating from L2/3 (top), L4 (middle), and L5/6 (bottom) of adult female or aged female animals. Right, Integration distance to each L2/3 cell originating from L2/3 (top), L4 (middle), and L5/6 (bottom) of adult male or aged male animals.

We next investigated if intracortical circuits to L2/3 pyramidal neurons change with aging [adult: *N* = 13 mice (7 females and 6 males); aged: *N* = 18 mice (6 females and 12 males)]. To visualize the connection pattern impinging on A1 L2/3 neurons, we performed whole-cell recordings from A1 L2/3 cells. We record from cells within a consistent region of A1 to avoid potential differences in rostrocaudal position. We then activated presynaptic neurons across the extent of A1 with LSPS. To isolate excitatory synaptic currents, we held cells at −70 mV (*E*_GABA_). Laser pulses were targeted to ∼900 distinct stimulus locations spanning all cortical layers around the recorded cell, and the resulting membrane currents were measured (Fig. 2*A*). We observed large, short-latency (<8 ms) inward currents when the stimulation location was close to the recorded neuron due to direct activation of the cell body and the proximal dendrites. In contrast, postsynaptic currents caused by activation of presynaptic neurons were long-latency (>8 ms) events (Meng et al., 2015, 2017a,b, 2020).

We mapped 105 L2/3 cells in A1 (48 cells in 13 young adult animals and 57 cells in 18 aged animals). For each cell, we identified stimulus locations that gave rise to an evoked EPSC and generated a binary input map. For each group, we aligned all maps to the soma position of the individual cells and averaged them (Fig. 2*B*), resulting in a spatial connection probability map for excitatory inputs. These maps allowed us to identify cortical locations that over the population gave rise to inputs to L2/3 neurons. Qualitatively comparing the connection probability maps, we observed that maps from adult and aged animals were similar (Fig. 2*B*).

To quantify the amount of input from each layer, we calculated the laminar changes of the connection properties of each cell as in previous studies (Meng et al., 2015, 2017a,b, 2020). We first visually identified layer boundaries in differential interference contrast images. We next quantified the amount of convergence from each layer to the L2/3 neurons. We calculated the total area within each layer from which EPSCs could be evoked by adding all the effective stimulation areas within each layer. A comparison of input areas indicates that the amount of intralaminar excitatory input from within L2/3 and from L4 and L5/6 is similar in aging and adult mice (Fig. 2*C*). In vivo imaging has revealed sex-specific differences between male and female mice with male mice showing a stronger effect of aging on sound-evoked responses and activity correlations (Shilling-Scrivo et al., 2021). We thus separately compared cells from male and female mice. We found that aging increased the excitatory input area from L2/3 and L5/6 in female but had no significant effects in male mice (Fig. 2*C*, Table 1). We also observed a trend toward decreased input evident also for inputs from L4 in males (Table 1). Thus, aging seems to affect the amount of ascending excitatory circuits to L2/3 neurons in female and male mice, but the sexes showed differences in what layer showed changes.

**Table 1.** Summary of changes for excitatory connections

Excitation			Adult		Aged		*p* values	
Input area (μm^2^)			Mean	Median	Mean	Median	KS test	Ranksum
	All	L2/3	1.84 × 10^4^	1.60 × 10^4^	2.23 × 10^4^	1.68 × 10^4^	0.68	0.57
		L4	7.96 × 10^3^	3.20 × 10^3^	6.37 × 10^3^	2.40 × 10^3^	0.96	0.97
		L5/6	1.04 × 10^4^	3.20 × 10^3^	1.00 × 10^4^	4.80 × 10^3^	0.49	0.19
			Mean	Median	Mean	Median	KS test	Kruskal–Wallis
	Female	L2/3	1.97 × 10^4^	1.60 × 10^4^	3.31 × 10^4^	2.88 × 10^4^	0.025	0.14
		L4	7.92 × 10^3^	1.60 × 10^3^	1.09 × 10^4^	8.00 × 10^3^	0.21	0.74
		L5/6	1.06 × 10^4^	1.60 × 10^3^	1.47 × 10^4^	8.80 × 10^3^	0.041	0.011
	Male	L2/3	1.73 × 10^4^	1.44 × 10^4^	1.30 × 10^4^	1.20 × 10^4^	0.44	1.0
		L4	8.00 × 10^3^	4.80 × 10^3^	2.46 × 10^3^	1.60 × 10^3^	0.22	0.16
		L5/6	1.02 × 10^4^	4.80 × 10^3^	5.94 × 10^3^	3.20 × 10^3^	0.25	1.0
Integration distance (μm)			Mean	Median	Mean	Median	KS test	Ranksum
	All	L2/3	191.1	174.5	198.6	210.0	0.17	0.23
		L4	113.0	100.0	124.5	98.0	0.96	0.53
		L5/6	191.9	214.1	223.1	272.0	0.36	0.30
			Mean	Median	Mean	Median	KS test	Kruskal–Wallis
	Female	L2/3	191.1	165.1	231.6	228.0	0.079	0.43
		L4	111.0	96.5	180.7	200.0	0.098	0.30
		L5/6	198.6	248.4	261.2	302.3	0.12	1.00
	Male	L2/3	191.1	189.9	172.9	173.0	0.36	1.00
		L4	114.7	100.0	80.6	0.0	0.35	1.00
		L5/6	186.2	196.7	193.3	234.1	0.87	1.00
Mean charge (pC)			Mean	Median	Mean	Median	KS test	Ranksum
	All	L2/3	1.28	1.26	0.95	1.12	0.26	0.078
		L4	0.54	0.95	0.42	0.59	0.074	0.023
		L5/6	0.45	0.38	0.44	0.39	0.99	0.94
			Mean	Median	Mean	Median	KS test	Kruskal–Wallis
	Female	L2/3	1.18	1.09	1.18	1.28	0.86	1.00
		L4	0.70	1.11	0.65	0.64	0.004	0.021
		L5/6	0.53	0.54	0.56	0.37	0.44	0.51
	Male	L2/3	1.35	1.28	0.77	0.86	0.028	0.025
		L4	0.40	0.64	0.24	0.46	0.14	1.00
		L5/6	0.38	0.35	0.35	0.40	0.63	1.00
Mean amplitude (pA)			Mean	Median	Mean	Median	KS test	Ranksum
	All	L2/3	28.55	28.57	28.08	30.48	0.81	0.57
		L4	17.13	30.38	19.94	28.95	0.56	0.78
		L5/6	18.21	23.15	22.38	25.11	0.96	0.73
			Mean	Median	Mean	Median	KS test	Kruskal–Wallis
	Female	L2/3	27.73	27.63	35.42	31.01	0.19	1.00
		L4	18.65	33.59	27.50	27.82	0.30	1.00
		L5/6	18.85	27.72	27.82	26.75	0.46	1.00
	Male	L2/3	29.25	29.48	22.34	29.54	0.76	1.00
		L4	15.85	25.36	14.03	32.01	0.59	1.00
		L5/6	17.98	23.09	18.13	21.61	0.70	1.00
Fractional input area (%)			Mean	Median	Mean	Median	KS test	Ranksum
	All	L2/3	63.50	63.15	60.13	60.00	0.076	0.64
		L4	16.80	10.80	12.00	12.50	0.22	0.53
		L5/6	19.71	13.27	27.88	22.22	0.047	0.085
			Mean	Median	Mean	Median	KS test	Kruskal–Wallis
	Female	L2/3	66.41	61.29	62.86	60.61	0.046	1.00
		L4	18.09	12.50	15.98	16.78	0.21	1.00
		L5/6	15.50	12.50	21.15	20.94	0.028	0.74
	Male	L2/3	60.84	65.00	57.70	56.76	0.83	1.00
		L4	15.61	9.09	8.45	5.00	0.47	0.93
		L5/6	23.55	21.43	33.85	30.00	0.89	1.00

Our areal measurement accounts for both changes in the input distribution within a layer, e.g., from L2 to L3, as well as changes in the orthogonal direction. Our thalamocortical slices preserve the macroscale rostrocaudally oriented tonotopic map in the slice plane. Therefore, the spatial extent of the input distribution along the rostrocaudal axis is a proxy for the integration along the tonotopic axis. To probe if the increase of L2/3 and L5/6 inputs in females occurred along the tonotopic axis, we next calculated the distance that includes 80% of the evoked EPSCs. We find that this intralaminar integration distance for inputs originating from all layers is unchanged in aged mice (Fig. 2*D*, Table 1), but opposing trends were visible in L4 between sexes. These results indicate that the same number of inputs originated from more cells across the same tonotopic area of L2/3 and L5/6 layer in old female mice and from L5/6 in male mice.

**Figure 3.**
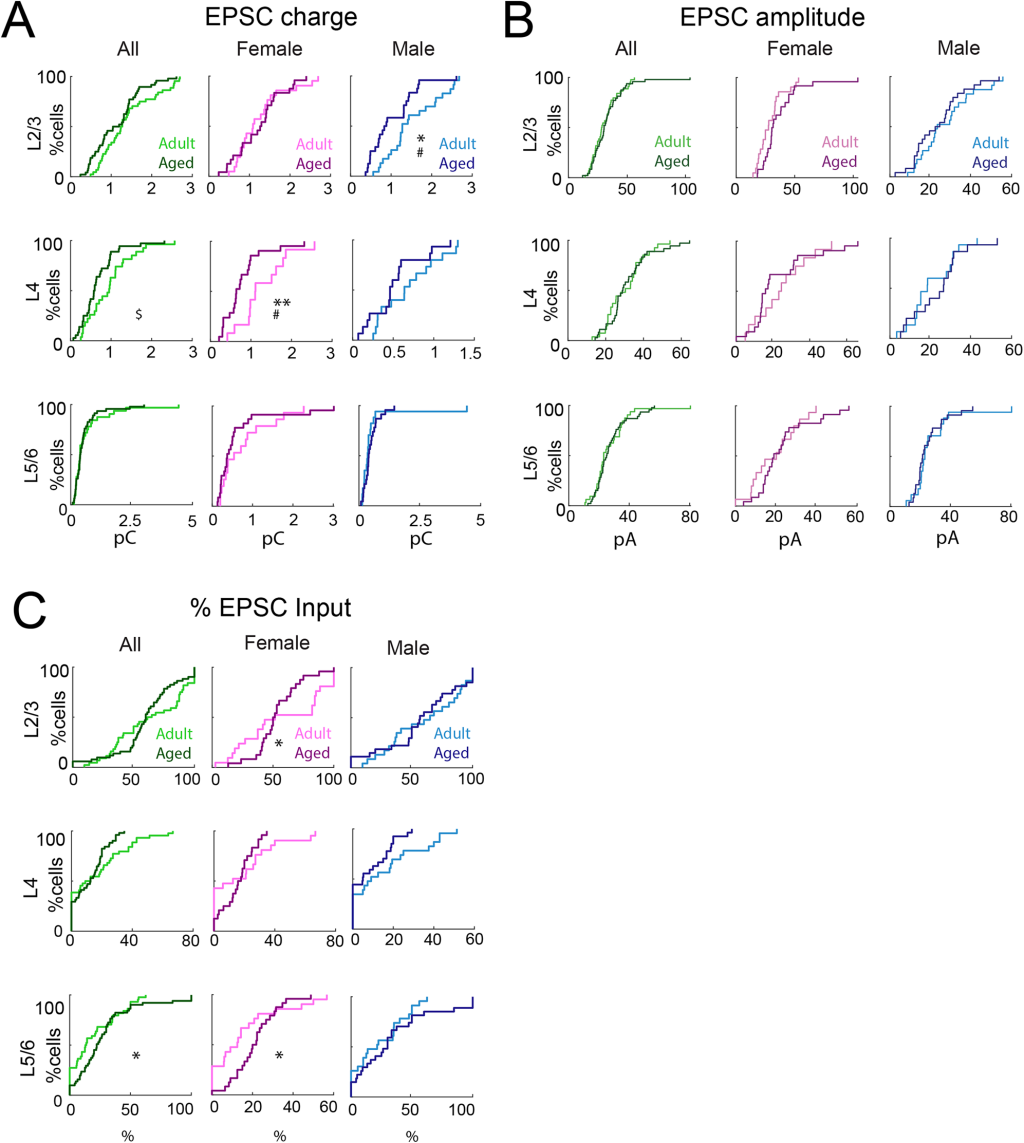
Sex-specific changes of connection strength and percentage inputs to A1 L2/3 neurons in aged mice. ***A***, Left, Distributions of mean EPSC charge of inputs originating from L2/3 (top), L4 (middle), and L5/6 (bottom) of young adult (light green) or aged (dark green) animals. ^$^*p*_Rank-sum_ < 0.05. Middle, Distribution of mean charge of inputs to each L2/3 cell originating from L2/3 (top), L4 (middle), and L5/6 (bottom) of adult female (light pink) or aged female (dark pink) animals. **p_KS_ < 0.01; ^#^*p*_Kruskal–Wallis_ < 0.05. Right, Distribution of mean charge of inputs to each L2/3 cell originating from L2/3 (top), L4 (middle), and L5/6 (bottom) of adult male (light blue) or aged male (dark blue) animals. *p_KS_ < 0.05; ^#^*p*_Kruskal–Wallis_ < 0.05. ***B***, Left, Distributions of mean EPSC amplitude of inputs originating from L2/3 (top), L4 (middle), and L5/6 (bottom) of young adult (light green) or aged (dark green) animals. Middle, Distribution of mean amplitude of inputs to each L2/3 cell originating from L2/3 (top), L4 (middle), and L5/6 (bottom) of adult female (light pink) or aged female (dark pink) animals. Right, Distribution of mean amplitude of inputs to each L2/3 cell originating from L2/3 (top), L4 (middle), and L5/6 (bottom) of adult male (light blue) or aged male (dark blue) animals. ***C***, Left, Distributions of fraction of total excitatory input originating from L2/3 (top), L4 (middle), and L5/6 (bottom) of young adult or aged animals. *p_KS_ < 0.05. Middle, Distributions of fraction of total excitatory input to each L2/3 cell originating from L2/3 (top), L4 (middle), and L5/6 (bottom) of adult female or aged female animals. *p_KS_ < 0.05. Right, Distributions of fraction of total excitatory input to each L2/3 cell originating from L2/3 (top), L4 (middle), and L5/6 (bottom) of adult male or aged male animals.

Functional circuit changes can occur through alteration of connection probabilities; that is, what fraction of cells receive inputs from a certain location. However, circuits can also change more subtly through changes of the connection strength between cells. Given that many synaptic proteins change with aging, we next tested if connection strength was altered. We measured the average size (transferred charge) of the evoked EPSCs and found that connections from L4 in old females showed lower synaptic strength (Fig. 3*A*, Table 1), while L2/3 inputs in old males were weakened (Fig. 3*A*, Table 1). Analysis of average amplitude of the evoked EPSCs showed no changes with age (Fig. 3*B*, Table 1). Thus, weakening of L2/3 inputs occurs with aging in males while weakening of L4 connections occur in females, but age had no effects on EPSC amplitude.

To compare how the laminar balance of inputs to every cell changed with age, we next computed the fraction of inputs originating from each layer by dividing the amount of input from each layer by the total amount of inputs. We found that cells from aging male mice received a similar fraction of inputs from all layers as young mice but that old female mice received an increased contribution from L2/3 and L5/6 (Fig. 3*C*, Table 1). These results indicate that L2/3 neurons in aged female mice tend to receive more interlaminar inputs, especially from L2/3 and L5/6.

Thus, these findings suggest that aged females have more excitatory inputs from L2/3 and L5/6, but weaker inputs from L4, while aged males show an unchanged connection pattern with weakened L2/3 inputs (Table 3). Together, these data show an altered and opposing contribution of L2/3 inputs in both aged males and females, but the mechanism of these changes differed between males and females. Thus, the circuit changes in A1 with aging vary by sex.

### L2/3 neurons in aged male A1 show reduced intralaminar inhibitory connections

**Figure 4.**
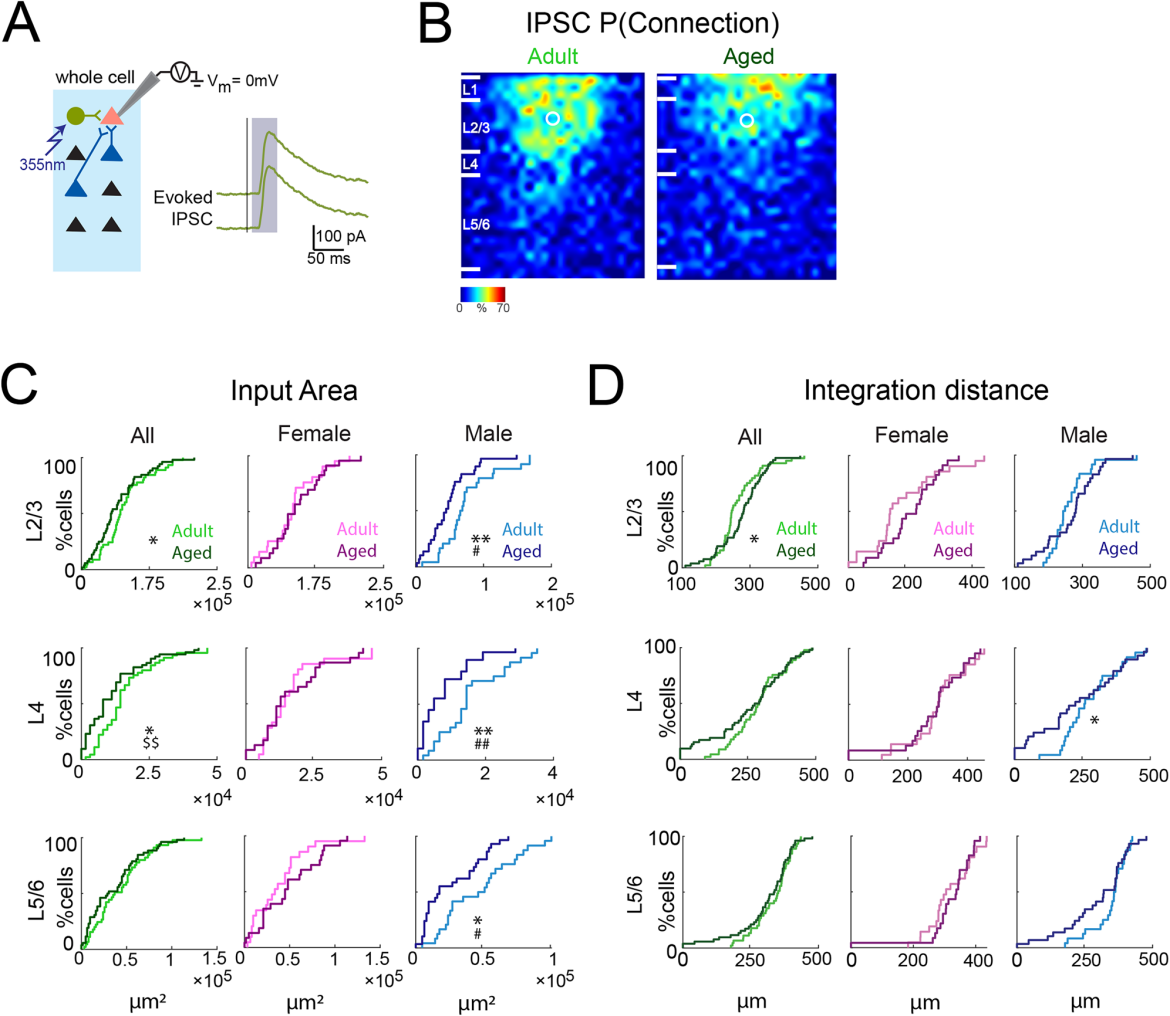
Sex-specific changes in inhibitory connections to A1 L2/3 neurons in aged male mice. ***A***, Schematic of LSPS when recording evoked IPSCs. Whole-cell voltage-clamp recordings were obtained from pink cell at holding potentials of 0 mV (IPSCs). If the presynaptic inhibitory neuron (e.g., green cells) synapses on the recorded neuron, an IPSC will be observed. Shown on the right are examplar patch-clamp recordings of IPSCs (green), acquired at the holding potential of 0 mV. Black vertical line indicates time of photostimulation; shaded gray box labels the time window of evoked responses. ***B***, Average maps of connection probability for inhibitory connections in young adult (left) and aged (right) animals. Maps are aligned to the soma location (white circle). Connection probability is encoded according to the pseudocolor scale. White horizontal lines indicate averaged laminar borders and are 100 μm long. ***C***, Left, Distributions of the area of inhibitory input originating from L2/3 (top), L4 (middle), and L5/6 (bottom) of young adult (light green) or aged (dark green) animals. *p_KS_ < 0.05; ^$$^*p*_Rank-sum_ < 0.01. Middle, Distributions of the area of inhibitory input originating from L2/3 (top), L4 (middle), and L5/6 (bottom) of adult female (light pink) or aged female (dark pink) animals. Right, Distributions of the area of inhibitory input originating from L2/3 (top), L4 (middle), and L5/6 (bottom) of young adult male (light blue) or aged male (dark blue) animals. *p_KS_ < 0.05; **p_KS_ < 0.01; ^#^*p*_Kruskal–Wallis_ < 0.05; ^##^*p*_Kruskal–Wallis_ < 0.01. ***D***, Left, Integration distance to each L2/3 cell originating from L2/3 (top), L4 (middle), and L5/6 (bottom) of young adult or aged animals. *p_KS_ < 0.05. Middle, Integration distance to each L2/3 cell originating from L2/3 (top), L4 (middle), and L5/6 (bottom) of adult female or aged female animals. Right, Integration distance to each L2/3 cell originating from L2/3 (top), L4 (middle), and L5/6 (bottom) of adult male or aged male animals. *p_KS_ < 0.05.

We next investigated inhibitory circuits by holding cells at 0 mV and performing LSPS. We recorded evoked IPSCs and derived inhibitory input maps (Fig. 4*A*). Averaging these maps yielded inhibitory connection probability maps for both groups. In contrast to the excitatory maps, the connection pattern of inhibitory circuits showed fewer connections from L2/3 and L4 in aged mice (Fig. 4*B*). Laminar analysis quantitatively supported these observations. L2/3 neurons from old mice received less inhibitory input from L2/3 and L4. However, we observed significant decrease in the inhibitory connections from all layers in male but not female mice (Fig. 4*C*, Table 2). However, the integration distance from all layers remains the same in terms of median between young and aged mice, suggesting that inhibitory inputs are originating from similar areas (Fig. 4*D*, Table 2). Together, our observations indicate that aging causes a reduction of inhibitory connections to L2/3 neurons in male mice.

**Table 2.** Summary of changes for inhibitory connections

Inhibition			Adult		Aged		*p* values	
Input area (μm^2^)			Mean	Median	Mean	Median	KS test	Ranksum
	All	L2/3	7.90 × 10^4^	7.20 × 10^4^	6.71 × 10^4^	5.52 × 10^4^	0.042	0.12
		L4	1.60 × 10^4^	1.44 × 10^4^	1.15 × 10^4^	8.00 × 10^3^	0.046	0.007
		L5/6	4.27 × 10^4^	3.68 × 10^4^	3.62 × 10^4^	2.96 × 10^4^	0.18	0.19
			Mean	Median	Mean	Median	KS test	Kruskal–Wallis
	Female	L2/3	8.23 × 10^4^	8.32 × 10^4^	9.16 × 10^4^	8.64 × 10^4^	0.95	1.00
		L4	1.65 × 10^4^	1.44 × 10^4^	1.68 × 10^4^	1.28 × 10^4^	0.69	1.00
		L5/6	3.77 × 10^4^	3.36 × 10^4^	4.87 × 10^4^	4.48 × 10^4^	0.65	0.91
	Male	L2/3	7.61 × 10^4^	6.72 × 10^4^	4.77 × 10^4^	4.48 × 10^4^	0.003	0.031
		L4	1.56 × 10^4^	1.44 × 10^4^	7.28 × 10^3^	4.80 × 10^3^	0.003	0.002
		L5/6	4.71 × 10^4^	4.88 × 10^4^	2.64 × 10^4^	1.76 × 10^4^	0.029	0.030
Integration distance (μm)			Mean	Median	Mean	Median	KS test	Ranksum
	All	L2/3	248.9	244.7	251.5	275.9	0.030	0.12
		L4	270.4	282.0	233.3	264.2	0.38	0.40
		L5/6	311.7	347.0	280.8	318.4	0.89	0.36
			Mean	Median	Mean	Median	KS test	Kruskal–Wallis
	Female	L2/3	257.3	245.5	262.5	275.9	0.092	1.00
		L4	286.0	299.2	263.1	298.0	0.94	1.00
		L5/6	305.1	301.1	295.4	335.4	0.66	1.00
	Male	L2/3	241.7	243.2	242.9	274.7	0.32	1.00
		L4	257.1	247.3	210.1	196.4	0.039	1.00
		L5/6	317.3	357.1	269.4	317.3	0.26	0.94
Mean charge (pC)			Mean	Median	Mean	Median	KS test	Ranksum
	All	L2/3	5.64	6.06	4.76	4.61	0.17	0.27
		L4	2.45	1.97	1.32	0.93	0.003	0.001
		L5/6	1.18	1.06	0.99	0.78	0.36	0.18
			Mean	Median	Mean	Median	KS test	Kruskal–Wallis
	Female	L2/3	5.46	5.45	0.18	5.71	0.92	1.00
		L4	2.97	2.20	1.47	1.69	0.15	0.56
		L5/6	1.17	1.06	0.95	0.96	0.97	1.00
	Male	L2/3	5.79	6.53	4.03	3.85	0.007	0.21
		L4	2.01	1.94	1.21	0.70	0.004	0.002
		L5/6	1.20	1.05	1.03	0.72	0.22	0.87
Mean amplitude (pA)			Mean	Median	Mean	Median	KS test	Ranksum
	All	L2/3	82.58	84.93	78.61	73.42	0.43	0.55
		L4	46.64	38.92	31.12	31.97	0.11	0.034
		L5/6	29.20	30.55	27.18	28.70	0.62	0.72
			Mean	Median	Mean	Median	KS test	Kruskal–Wallis
	Female	L2/3	76.49	83.77	98.75	98.11	0.30	0.86
		L4	52.17	36.84	37.48	39.91	0.80	1.00
		L5/6	27.32	28.49	28.51	34.50	0.046	0.42
	Male	L2/3	87.72	90.28	62.88	62.88	0.080	0.055
		L4	41.96	39.32	26.15	26.15	0.032	0.094
		L5/6	30.79	30.70	26.14	26.14	0.009	0.047
Fractional percentage input area (%)			Mean	Median	Mean	Median	KS test	Ranksum
	All	L2/3	56.68	58.14	60.72	57.51	0.36	0.33
		L4	12.35	10.29	9.44	9.73	0.15	0.083
		L5/6	30.98	30.56	29.84	32.36	0.55	0.77
			Mean	Median	Mean	Median	KS test	Kruskal–Wallis
	Female	L2/3	58.73	58.14	62.37	58.23	0.13	1.00
		L4	13.13	11.94	9.90	10.81	0.48	1.00
		L5/6	28.15	29.93	27.74	33.63	0.28	1.00
	Male	L2/3	54.88	56.95	59.42	56.76	0.26	1.00
		L4	11.66	9.18	9.08	7.94	0.078	1.00
		L5/6	33.45	31.04	31.51	32.05	0.26	1.00

**Figure 5.**
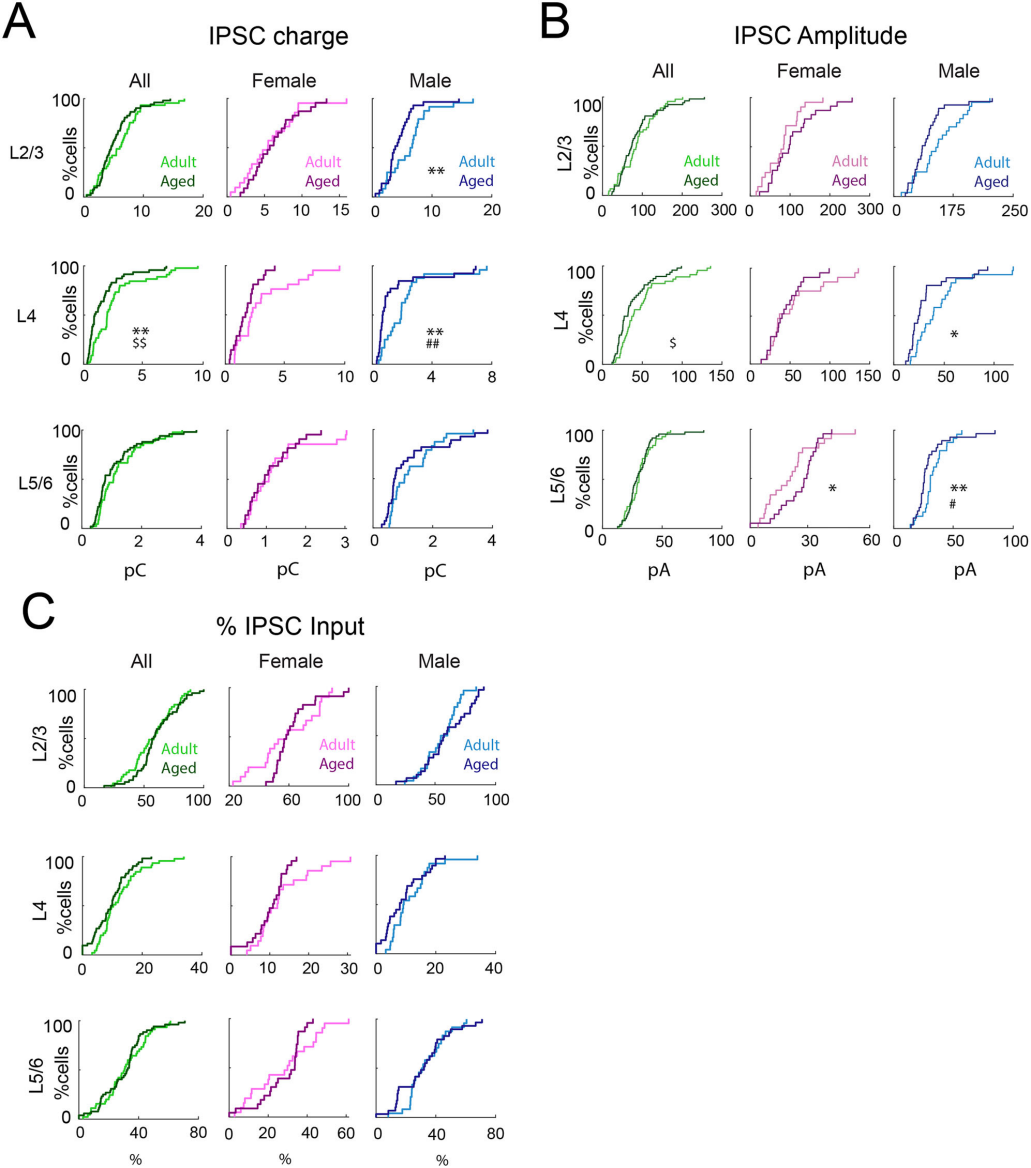
Inhibitory connection strength and percentage of inhibitory inputs originating from each layer remain unchanged between young and aged animals. ***A***, Left, Distributions of mean IPSC charge of inputs originating from L2/3 (top), L4 (middle), and L5/6 (bottom) of young adult (light green) or aged (dark green) animals. **p_KS_ < 0.01; ^###^*p*_Kruskal–Wallis_ < 0.001. Middle, Distribution of mean charge of inputs to each L2/3 cell originating from L2/3 (top), L4 (middle), and L5/6 (bottom) of adult female (light pink) or aged female (dark pink) animals. ^#^*p*_Kruskal–Wallis_ < 0.05. Right, Distribution of mean charge of inputs to each L2/3 cell originating from L2/3 (top), L4 (middle), and L5/6 (bottom) of adult male (light blue) or aged male (dark blue) animals. **p_KS_ < 0.01; ^#^*p*_Kruskal–Wallis_ < 0.05. ***B***, Left, Distributions of mean IPSC amplitude of inputs originating from L2/3 (top), L4 (middle), and L5/6 (bottom) of young adult or aged animals. ^$^*p*_Rank-sum_ < 0.05. Middle, Distributions of mean amplitude of inputs to each L2/3 cell originating from L2/3 (top), L4 (middle), and L5/6 (bottom) of adult female or aged female animals. *p_KS_ < 0.05. Right, Distributions of fraction of mean amplitude of inputs to each L2/3 cell originating from L2/3 (top), L4 (middle), and L5/6 (bottom) of adult male or aged male animals. *p_KS_ < 0.05; **p_KS_ < 0.01; ^#^*p*_Kruskal–Wallis_ < 0.05. ***C***, Left, Distributions of fraction of total inhibitory input originating from L2/3 (top), L4 (middle), and L5/6 (bottom) of young adult or aged animals. Middle, Distributions of fraction of total inhibitory input to each L2/3 cell originating from L2/3 (top), L4 (middle), and L5/6 (bottom) of adult female or aged female animals. Right, Distributions of fraction of total inhibitory input to each L2/3 cell originating from L2/3 (top), L4 (middle), and L5/6 (bottom) of adult male or aged male animals.

Previous studies showed that the expression of rate-limiting enzymes, GAD65 and GAD67, which catalyze the conversion of glutamate to GABA, are decreased in aging in both IC and A1 (Milbrandt et al., 2000; Burianova et al., 2009), suggesting that strength of inhibition could be altered in aged mice. To test this hypothesis, we calculated the average charge of IPSCs. We found that IPSCs evoked from L2/3 and L4 in aged male were weaker than in young mice (Fig. 5*A*, Table 2). We also observed a trend toward decreased input strength from L4 in aged females (Fig. 5*A*, Table 2). Analysis of average amplitude of IPSCs yielded similar result (Fig. 5*B*, Table 2). Thus, aging reduces the transferred inhibitory connection strength dependent of sex (Fig. 5*A*,*B*).

We next computed the relative amount of inhibitory input from each layer and found that this was unchanged with age (Fig. 5*C*), indicating that inhibitory inputs from all layers were reduced with a similar proportion in male mice with aging. Thus, our findings showed a reduction in the numbers of inhibitory inputs to L2/3 and L4 cells in old male mice (Table 3).

**Table 3.** Summary of connection changes

		Area		Distance		Charge		% input	
		M	F	M	F	M	F	M	F
Excitation	L2/3	o	+	o	o	−	o	o	+
	L4	o	o	o	o	o	−	o	o
	L5/6	o	+	o	o	o	o	o	+
Inhibition	L2/3	−	o	o	o	o	o	o	o
	L4	−	o	o	o	−	o	o	o
	L5/6	−	o	o	o	o	o	o	o

### Excitation/inhibition balance in female mice is altered by aging

**Figure 6.**
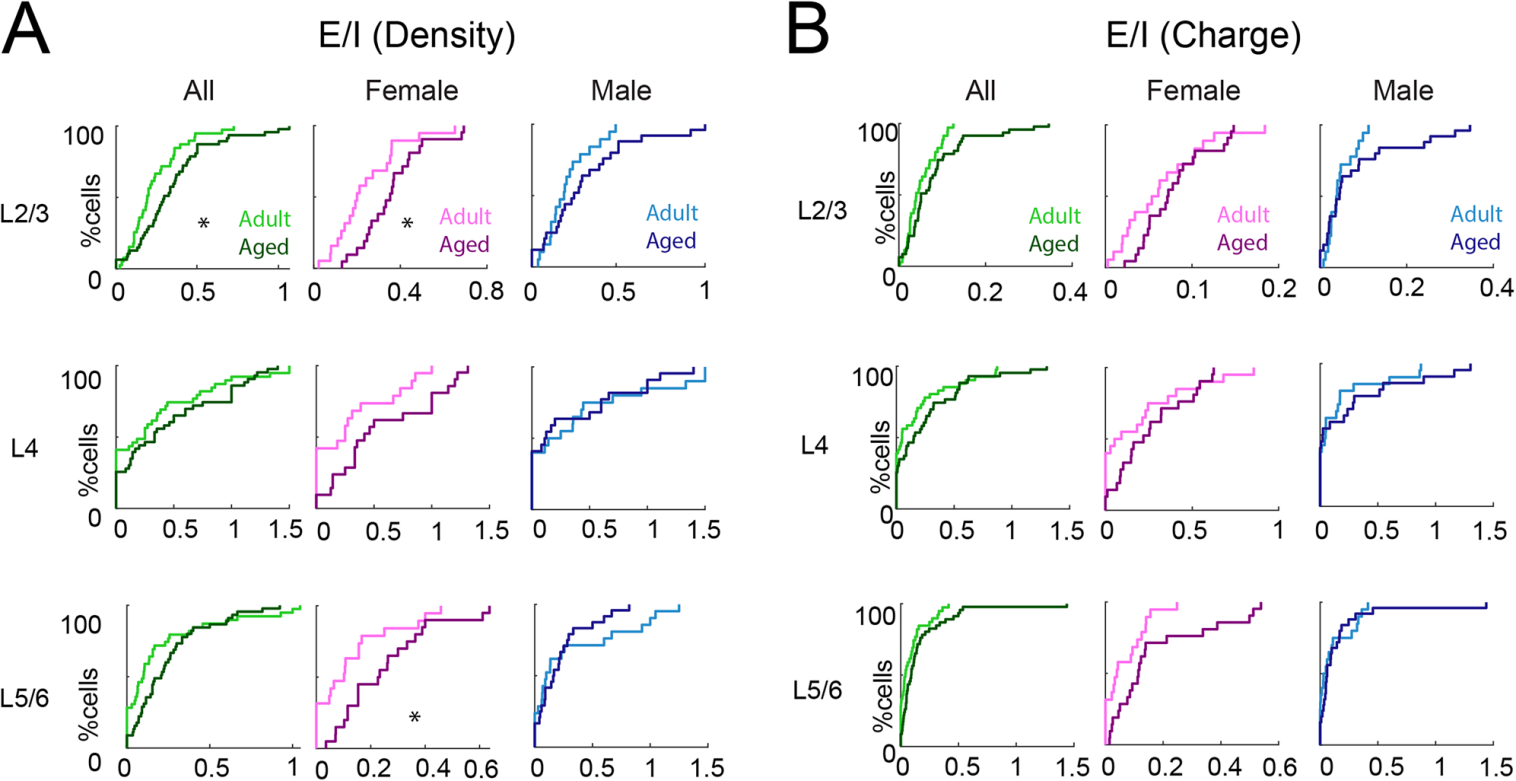
Aged female animals show altered *E*/*I* ratio. ***A***, Left, Distributions of *E*/*I* based on input numbers (density) from L2/3 (top), L4 (middle), and L5/6 (bottom) of young adult (light green) or aged (dark green) animals. *p_KS_ < 0.05. Middle, Distributions of *E*/*I* based on input numbers (density) to each L2/3 cell originating from L2/3 (top), L4 (middle), and L5/6 (bottom) of adult female (light pink) or aged female (dark pink) animals. *p_KS_ < 0.05. Right, Distributions of *E*/*I* based on input numbers (density) from L2/3 (top), L4 (middle), and L5/6 (bottom) of adult male (light blue) or aged male (dark blue) animals. ***B***, Left, Distributions of *E*/*I* based on transferred charge originating from L2/3 (top), L4 (middle), and L5/6 (bottom) of young adult or aged animals. Middle, Distributions of *E*/*I* based on transferred charge to each L2/3 cell originating from L2/3 (top), L4 (middle), and L5/6 (bottom) of adult female or aged female animals. Right, Distributions of *E*/*I* based on transferred charge to each L2/3 cell originating from L2/3 (top), L4 (middle), and L5/6 (bottom) of adult male or aged male animals.

The concomitant occurrence of synaptic excitation and inhibition maintains balance between excitation and inhibition. In all sensory cortices, excitation and inhibition wax and wane together when responding to sensory stimuli (Anderson et al., 2000; Swadlow, 2003; Wehr and Zador, 2003; Tan et al., 2004; Wilent and Contreras, 2005), and the balance of these two opposing forces is critical for proper cortical function (Sohal and Rubenstein, 2019). Given that the *E*/*I* ratio changes and is crucial during development (Hensch and Fagiolini, 2005; Chu and Anderson, 2015; Hu et al., 2017), we asked whether *E*/*I* balance also changes during the aging process. For each cell, we calculated the *E*/*I* balance of the inputs from each layer based on the spatial connection probability and the charge of PSCs as in our prior studies (Meng et al., 2015). We found that the *E*/*I* ratio based on density was unchanged in aged females but showed a trend toward excitation (Fig. 6*A*; L2/3: adult: mean: 0.24, median: 0.20; aged: mean: 0.36, median: 0.35, *p*_KS_ = 0.036, *p*_Kruskal–Wallis_ = 0.11. L4: adult: mean: 0.30, median: 0.25; aged: mean: 0.57, median: 0.42, *p*_KS_ = 0.10, *p*_Kruskal–Wallis_ = 0.19. L5/6: adult: mean: 0.13, median: 0.10; aged: mean: 0.25, median: 0.23, *p*_KS_ = 0.046, *p*_Kruskal–Wallis_ = 0.10). *E*/*I* ratios based on charge yielded similar results for input originating in L5/6 in aged female animals (Fig. 6*B*). There was no significant change in *E*/*I* ratios for male animals. These results indicate that as the relative number of intralaminar inhibitory inputs to L2/3 neurons decreased in old male mice, so did excitatory inputs. In contrast, the increase in excitatory inputs in aged female mice was not matched by changes in inhibitory inputs.

### Decreased circuit similarity in aged mice

**Figure 7.**
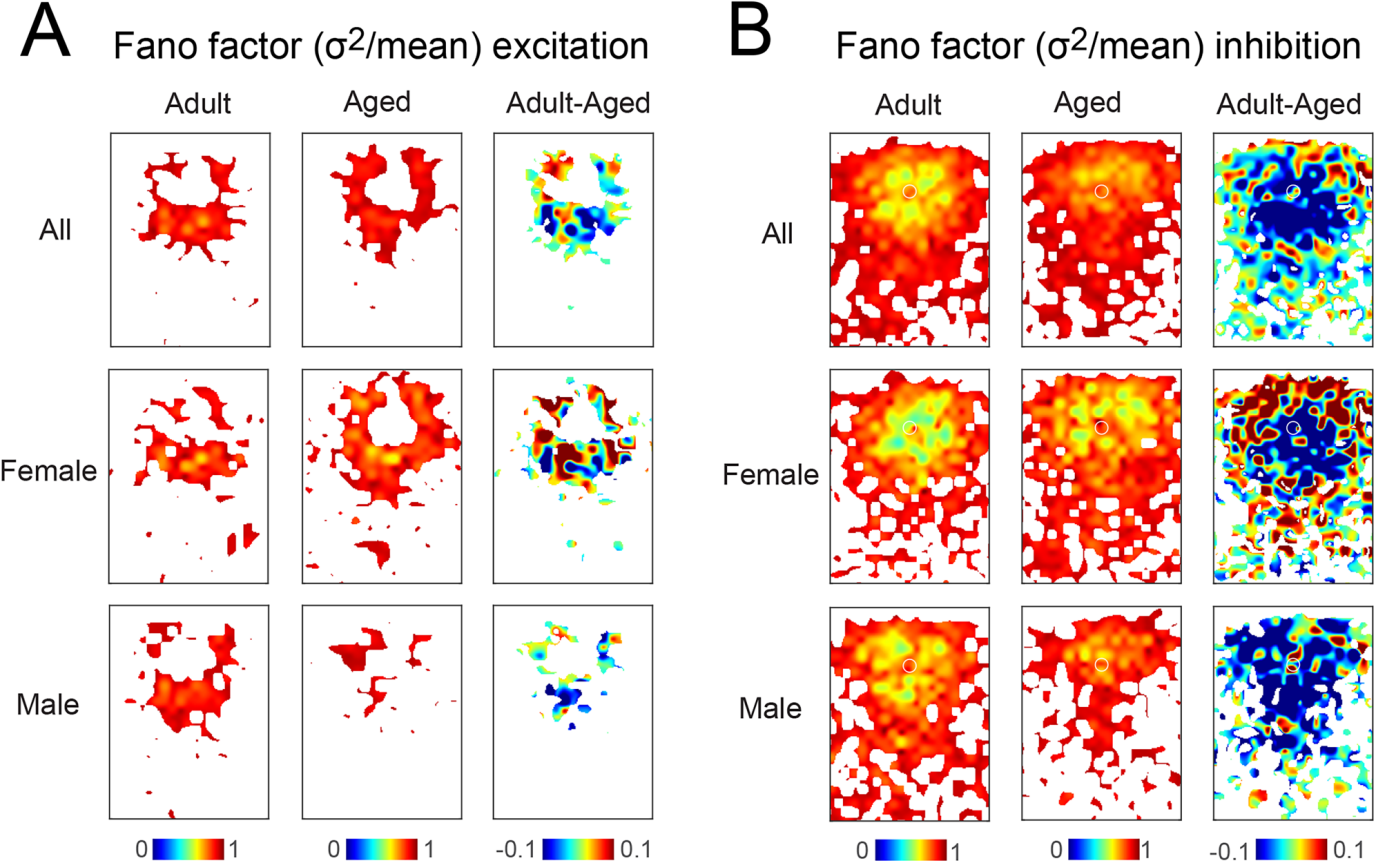
Circuit similarity decreased in aged mice. Fano factors (variance/mean) for excitatory connection maps of adult (left), aged (middle), and the differences between adult and aged (right). The Fano factor is represented in pseudocolor. The smaller the Fano factor, the greater the similarity in the population. In the maps, white represents areas where the probability of the connections was <5% and a Fano factor was not calculated. ***A***, Fano factors for the excitatory connection maps. ***B***, Fano factors for the inhibitory connection maps.

L2/3 cells in adult A1 show heterogeneity in their functional interlaminar circuits (Meng et al., 2017b). This heterogeneity emerges over development, coinciding with a decrease in functional activity correlations (Meng et al., 2020). To gain further insight into the diversity of the spatial circuit patterns, we calculated the Fano factor (FF = *σ*^2^/*μ*) of the spatial connection probability map. Thus, while Figure 2 shows the average input probability for each spatial location in each group of cells, we here also consider the variability at each location. To calculate the Fano factor for each spatial location, we divide the standard deviation of the input probability by the mean of the input probability. A low Fano factor indicates that within the population, most cells received similar inputs from a particular location, whereas a high Fano factor indicates that there is a large variability in the amount of input cells received from a particular location. We then calculated the difference between the Fano factor in adult and aged mice. For excitatory inputs, the difference in the Fano factor was negative with age for inputs originating in L4 in columnar locations below the recorded L2/3 cell in both male and female mice (Fig. 7*A*). This indicates that the Fano factor at these locations was larger in the aged male mice than in adult male mice. However, aging females also showed patchy areas of increased and decreased Fano factor in L2/3. This analysis indicates an increased variability in where L4 inputs originated with aging. In contrast, for inhibitory inputs in both males and females, there is a decrease in Fano factor within L2/3 at proximal locations around the L2/3 soma (Fig. 7*B*). Thus, inhibition from nearby cells becomes more variable with age. In females, there is also an increase in the Fano factor for more distal inputs from L2/3. In addition, in males there is a decrease in Fano factor for inhibitory inputs from L4. Thus, aging increases the diversity of ascending excitatory intracortical circuits to L2/3 neurons in both females and males, as well as the short-range inhibitory intralaminar inputs. These effects are most extensive in male mice.

**Figure 8.**
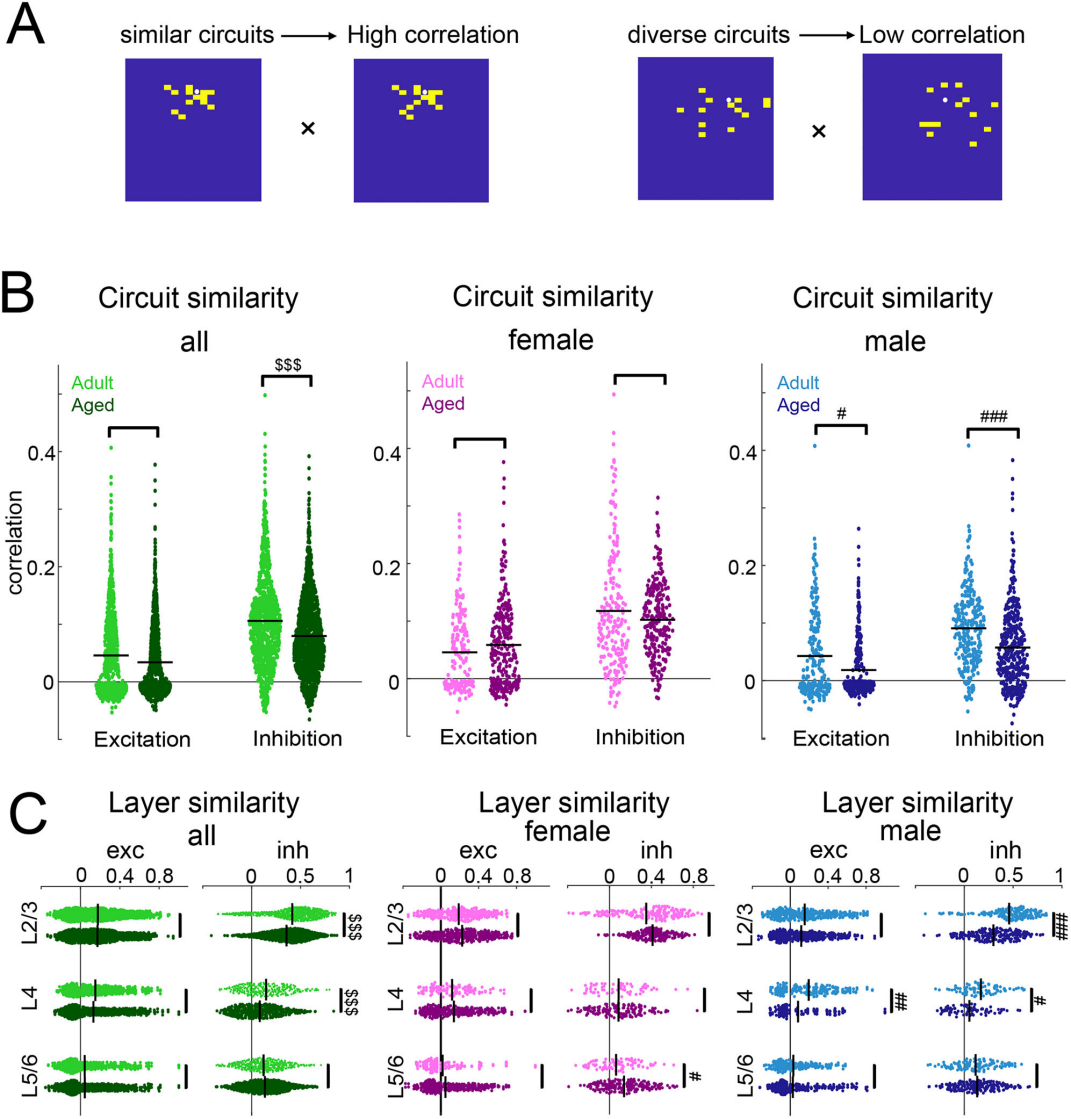
Decreased similarity of input maps in aged mice. ***A***, Schematic illustration of how pairwise correlation between two maps is calculated. Filled white circle represents recorded neuron. A yellow square represents a stimulus location that has monosynaptic connection to the recorded cell; a blue square represents a location with no connection to the recorded cell. For the pairwise correlation calculation, yellow and blue squares are assigned values of 1 and 0, respectively. ***B***, Correlation of excitatory and inhibitory circuit patterns across all layers. Left, Correlation of circuit patterns for all adult (light green) and aged (dark green) animals. ^$$$^*p*_Rank-sum_ < 0.001. Middle, Correlation of circuit patterns for adult female (light pink) and aged female (dark pink) animals. Right, Correlation of circuit patterns for adult male (light blue) and aged male (dark blue) animals. ^#^*p*_Kruskal–Wallis_ < 0.05; ^###^*p*_Kruskal–Wallis_ < 0.001. ***C***, Correlation of circuit patterns within L2/3 (left), L4 (middle), and L5/6 (right). Left, Circuit similarity from different layers in adult and aged animals. ^$$$^*p*_Rank-sum_ < 0.001. Middle, Circuit similarity from different layers in adult and aged female animals. ^#^*p*_Kruskal–Wallis_ < 0.05. Right, Circuit similarity from different layers in adult and aged male animals. ^#^*p*_Kruskal–Wallis_ < 0.05; ^##^*p*_Kruskal–Wallis_ < 0.01; ^###^*p*_Kruskal–Wallis_ < 0.001.

To examine whether aging alters the circuit heterogeneity of L2/3 cells, we calculated the correlation between individual connection maps from all recorded cells (Fig. 8*A*). We aligned the connection maps of recorded neurons within each group to their soma positions and compared the connection patterns between cells. This analysis showed that the correlation of excitatory and inhibitory circuits decreased in old male mice while the similarity of inhibitory circuits trended lower in old female mice (Fig. 8*B*, male excitation: adult: mean: 0.0427, median: 0.00738; aged: mean: 0.0183, median: −0.00483; *p*_Kruskal–Wallis_ = 0.031. inhibition; adult: mean: 0.0908, median: 0.0911; aged: mean: 0.0573, median: 0.0448; *p*_Kruskal–Wallis_ < 0.001). In male mice the decrease in circuit similarity was driven by decreases in both L4 and L2/3 (Fig. 8*C*; L2/3: adult mean: 0.460, median: 0.498; aged mean: 0.299, median: 0.307, *p*_Kruskal–Wallis_ < 0.001. L4: adult mean: 0.170, median: 0.147; aged mean: 0.0524, median: 0.0390; *p*_Kruskal–Wallis_ = 0.002). Thus, aging increases the diversity of intracortical circuits to L2/3 neurons, and these effects are most pronounced in male mice. Together, these results indicate that aging changes not only the amount and strength of inputs but also the spatial functional connection pattern of inputs to L2/3 neurons.

## Discussion

Our main results show a hypoconnectivity of intra- and interlaminar inhibitory connections to A1 L2/3 due to aging predominantly in male CBA mice. The circuit changes we observe are consistent with altered functional responses in A1 of aged CBA mice, which are more apparent in aged male mice.

We observed a reduction in the strength of intralaminar excitatory inputs within L2/3 cells with age in male mice and a reduction in excitatory strength of interlaminar inputs from L4 in female mice. While prior work did not examine sex differences, these results are consistent with the observation of reduced dendritic spine density in the aged brain (Dickstein et al., 2013; Hickmott and Dinse, 2013). MGB cells, which provide strong input to L4 but also weaker input to L2/3 (Ji et al., 2016), in aged animals show hyperexcitability due to reduced tonic inhibition (Richardson et al., 2013), possibly increasing thalamocortical drive. Assuming a homeostatic framework, the decreased inter- and intralaminar cortical connection strength to L2/3 could be a compensation mechanism for increased ascending drive from thalamocortical recipient layers or from higher-order thalamic nuclei (Zhang and Bruno, 2019). We here measure the connection strength between cells. These connections could be mediated by changes in the synaptic strength and release probability of individual synapses or the number of synaptic contacts between cells. Our LSPS method cannot distinguish between these possibilities. The changes in connection strength are consistent with a loss of synaptic AMPA receptors and hypofunction of NMDA receptors that have been observed in the aged hippocampus and in other sensory areas (Gocel and Larson, 2013; Kumar et al., 2019).

Taken together, our results suggest that the aged A1 in CBA mice shows reduced intra- and interlaminar excitation. The decreased strength of excitatory inputs we observe in female mice might contribute to the reduced bandwidth of aging L2/3 neurons (Shilling-Scrivo et al., 2021) as L4 neurons show broader tuning than L2/3 neurons (Winkowski and Kanold, 2013; Bowen et al., 2020). We find that the distribution of integration bandwidth of inhibitory inputs is altered with aging in male mice with more cells receiving less inhibitory inputs. Given the ∼300 µm/oct tonotopic slope in A1 (Bandyopadhyay et al., 2010; Guo et al., 2012), this decrease could lead to decreased integration bandwidth and thus possibly to decreased suppression of wideband backgrounds consistent with in vivo results (Shilling-Scrivo et al., 2021).

Much focus has been placed on the changes in inhibition during aging (Chao and Knight, 1997; Caspary et al., 1999, 2008, 2013; Kok, 1999; Ling et al., 2005; Stanley et al., 2012; Rozycka and Liguz-Lecznar, 2017; Recanzone, 2018; Rogalla and Hildebrandt, 2020). Indeed, we did observe a reduction of functional inhibitory connections, but this was only present in male mice. The interneuron population is stable with aging (Ouellet and de Villers-Sidani, 2014; Burianova et al., 2015; Rogalla and Hildebrandt, 2020) and no age-related changes in the overall number, total binding, or affinity of GABA_A_ receptors have been observed (Heusner and Bosmann, 1981; Komiskey and MacFarlan, 1983; Reeves and Schweizer, 1983; Komiskey, 1987). Together with our data this points to a loss of inhibitory connections in male mice. However, prior studies did not separate animals by sex; thus it is possible, for example, that changes in cell number exist in aged male mice.

We found that the inhibitory input area originating from L4 is more concentrated in aged male animals and overall inhibition is more diverse between cells. Inhibition is critical for shaping tuning properties and information redundancy (Sillito, 1977, 1979; Kyriazi et al., 1996; Wang et al., 2000, 2002; Kurt et al., 2006; Razak and Fuzessery, 2009; Sun et al., 2010; Gaucher et al., 2013; Li et al., 2014; Zhou et al., 2014). Decreased inhibition in male mice could contribute to the increased population correlations observed in vivo (Shilling-Scrivo et al., 2021). Moreover, we observe an increased diversity in proximal inhibition from within L2/3 in both male and female mice, suggesting that inhibitory circuits that provide local inhibition might be a target of aging. Local parvalbumin (PV) neurons provide input to L2/3 cells (Tasaka et al., 2023), and inhibition by PV cells are thought to be decreased in aging (Stanley et al., 2012; Ouellet and de Villers-Sidani, 2014; Brewton et al., 2016). Thus, we speculate that the decreased and more diverse intralaminar inhibition is due to loss of PV input. In female mice we see an increased similarity in distal intralaminar inputs. We speculate that this reflects a compensatory upregulation of inhibitory inputs from other sources, e.g., somatostatin (SST) neurons (Tasaka et al., 2023). Our results suggest changes in inhibitory circuits. While LSPS has the great advantage of high throughput, it lacks target specificity; thus future experiments (e.g., using selective Cre lines) are needed to disentangle the differential contributions of different cell classes during aging. However, a potential confound for such studies is that most transgenic mouse lines exist on the C57BL/6 strain, which suffers early loss of high-frequency hearing. Here, we study mice of the CBA strain, which do not suffer from early high-frequency hearing loss (Willott et al., 1988, 1991; Frisina et al., 2011; Bowen et al., 2020; Shilling-Scrivo et al., 2021), as mice of the common C57BL/6 strain do (Henry and Chole, 1980; Willott, 1986; Spongr et al., 1997). In aged C57BL/6 mice, functional hypoconnectivity of both excitatory and inhibitory circuits was present (Xue et al., 2023). Given that we observe changes to the number of inhibitory circuits and the diversity of both excitatory and inhibitory circuits, the effects of aging on central circuits might first be an effect on circuit diversity followed by a loss of circuits. The mechanisms underlying the control of circuit diversity are unknown. However, it is reasonable to assume that circuit diversity likely is a result of functional plasticity, especially given that during development, diversity emerges during the critical period (Meng et al., 2020). Thus, we speculate that activity-dependent plasticity mechanisms that also aid in stabilizing networks might be dysfunctional in aging A1 in a sex-dependent manner. Indeed, behavioral training, which likely engages such mechanisms, is able to rescue many of the behavioral age-related hearing deficits (Cheng et al., 2017, 2020; Jendrichovsky et al., 2024; Mittelstadt et al., 2024).

A less dispersive input area from L4 might contribute to an abnormal tuning bandwidth (Turner et al., 2005; Shilling-Scrivo et al., 2021). We find that excitation from L5/6 is increased in aged female animals. Inputs from L5/6 are thought to be important in gain control (Chambers et al., 2016; Onodera and Kato, 2022); thus strengthening of these inputs could also contribute to the increased population correlations observed in vivo (Shilling-Scrivo et al., 2021).

Our results show that the *E*/*I* balance in male animals remains stable with aging. This suggests a coordinated change of both excitation and inhibition. However, in females we observe an increasing trend in *E*/*I* due to increased excitation from L2/3 and L5/6. While an *E*/*I* imbalance has been found in the parietal cortex with age-related cognitive impairment, aged animals with normal cognition do not show an imbalance (Wong et al., 2006), consistent with our results. We are using voltage clamp, and even with Cs^+^ in our electrode recording solution, distal dendrites will not be clamped at the holding potential. Thus, we might miss inputs targeting these dendritic locations and underestimate the excitatory inputs to the recorded cells.

Aging can alter the tonotopic map in A1 with aged animals showing a higher degree of scatter in frequency tuning and disorganized frequency distribution (de Villers-Sidani et al., 2010; Kamal et al., 2013). Meanwhile, aged animals show more diverse A1 receptive field possibly due to dysregulated plasticity in the aging cortex (Turner et al., 2005; Cisneros-Franco et al., 2018). In contrast, groups of cells in aged animals show increased functional correlations (Shilling-Scrivo et al., 2021). These results suggest a more heterogeneous functional organization but more homogenous activation of aged A1 neurons. These results are consistent with our observation of increased diversity of intracortical circuits and changes in intracortical inhibitory circuits. Moreover, in addition to intracortical circuit changes, functional changes might also reflect alterations in axonal bouton dynamics that are present in aged thalamocortical axons (Grillo et al., 2013).

We find a strong effect of sex on the age-related changes in A1 circuits in that changes to inhibition are most pronounced in males but that distinct changes exist in females. We here investigated mice <24 months of age. We speculate that central aging in males might be accelerated compared with females (Henry, 2004). This effect of sex we reveal is consistent with in vivo studies that show that male CBA mice have much larger age-related increases in correlated activity and behavioral changes than females (Shilling-Scrivo et al., 2021, 2022; Mittelstadt et al., 2024). Male grasshopper mice also show larger increase in auditory brainstem responses threshold with age than females (Kobrina et al., 2021). These results strongly suggest that much more work needs to be done in humans and other model species to see if this extends beyond rodents (Nolan, 2020). Indeed, already sex differences have been found in human cortical synaptic connections (Alonso-Nanclares et al., 2008), and, in humans, males have larger age-dependent decreases in hearing than females (Pearson et al., 1995; Cruickshanks et al., 1998; Helzner et al., 2005; Nolan, 2020; Hansen et al., 2024). Moreover, given that we see distinct differences in the age-related changes between males and females, our data suggest that therapeutic approaches, e.g., training paradigms (Jendrichovsky et al., 2024; Mittelstadt et al., 2024), may need to be tailored by sex.

Taken together, our findings delineate sex-dependent effects of aging on mouse primary auditory cortex on a microcircuit level. We demonstrate that aging has more dramatic effects on A1 in male CBA mice, resulting mostly in decreased inhibitory circuits in aged males. Our results suggest that the altered response properties to sound in aged male CBA A1 are mostly due to the changes of inhibitory connections but that changes in excitatory connections might contribute.

## Synthesis

Reviewing Editor: Christine Portfors, Washington State University Decisions are customarily a result of the Reviewing Editor and the peer reviewers coming together and discussing their recommendations until a consensus is reached. When revisions are invited, a fact-based synthesis statement explaining their decision and outlining what is needed to prepare a revision will be listed below. The following reviewer(s) agreed to reveal their identity: NONE. Note: If this manuscript was transferred from JNeurosci and a decision was made to accept the manuscript without peer review, a brief statement to this effect will instead be what is listed below.

Thank you for addressing all the previous reviewers' comments.
